# Supplementary material for: On CA1 ripple oscillations in rats and the reassessment of asynchronicity evidence
Source: eLife. 2025 Dec 17;14:RP106201. doi: 10.7554/eLife.106201 (PMC12711197; doi:10.7554/eLife.106201)
Supplement: Supplementary file 1 [file elife-106201-supp1.zip › SchefferTeixeira_Supplementary_File_1.odt]

Supplementary File 1 - Statistical table summarising all statistical comparisons carried out in the study.

						

| Figure | Metric | Variables | Sample Size | Statistical Test | Confidence Intervals for the model coefficient |
| --- | --- | --- | --- | --- | --- |
| 2A left | Ripple abundance (event counts per second) for left and right hippocampi. | Response: Counts per second: quantitative continuous. Explanatory: (1) Side: categorical nominal with two levels: left and right. Left was set as reference. Only threshold = 2 std was tested, since it included ripple events for all other thresholds. | Nleft = 50 Nright = 40 | Linear Mixed Model Regression Side Coef. = 0.012 z = 1.44 P-value = 0.15 TOST Bound = +- 0.032 p-value = 0.0084 | Side IC95% = [-0.004, 0.029] |
| 2A right | Hedges’ g effect size for ripple abundance. | Effect size difference right minus left. | Nleft = 50 Nright = 40 | Permutation t-test Difference = 0.082301 P-value = 0.7 | IC95% = [-0.33, 0.5] |
| 2B | Ripple abundance (event counts per second) for left and right hippocampi using different threshold levels. | Response: Counts per second: quantitative continuous. Explanatory: (1) Side: categorical nominal with two levels: left and right. Left was set as reference. (2) Detection threshold: quantitative discrete with levels from 2 to 10. | Nleft = 50 Nright = 40 with repeated measures across thresholds. | Linear Mixed Model Regression Side Coef. = 0.025 z = 1.422 p-value = 0.155 Threshold Coef. = -0.051 z = -28.350 p-value = 8.33 x 10-177 Side x Threshold Coeff = -0.003 z = -1.094 p-value = 0.274 | Side IC95% = [-0.009, 0.059] Threshold IC95% = [-0.054, -0.047] Side x Threshold IC95% = [-0.008, 0.002] |
| 2C left | Ripple mean frequency for left and right hippocampi. | Response: Frequency: quantitative continuous. Explanatory: (1) Side: categorical nominal with two levels: left and right. Left was set as reference. | Nleft = 50 Nright = 40 | Linear Mixed Model Regression Side Coef. = -0.907 z = -0.801 P-value = 0.42 TOST Bound = +- 4.87 p-value = 0.0076 | Side IC95% = [-3.124, 1.311] |
| 2C right | Hedges’ g effect size for mean ripple frequency. | Effect size difference right minus left. | Nleft = 50 Nright = 40 | Permutation t-test Difference = 0.074833 P-value = 0.73 | IC95% = [-0.337, 0.466] |
| 2E left | Inter-ripple intervals (in seconds) for left and right hippocampi. | Response: Time: quantitative continuous. Explanatory: (1) Side: categorical nominal with two levels: left and right. Left was set as reference. | Nleft = 50 Nright = 40 | Linear Mixed Model Regression Side Coef. = -0.048 z = -1.505 P-value = 0.132 TOST Bound = +- 0.12 p-value = 0.012 | Side IC95% = [-0.111, 0.015] |
| 2E right | Hedges’ g effect size for inter-ripple intervals. | Effect size difference right minus left. | Nleft = 50 Nright = 40 | Permutation t-test Difference = -0.114 P-value = 0.6 | IC95% = [-0.514, 0.29] |
| 2G left | Mean number of cycles per ripple event for left and right hippocampi. | Response: Mean cycle counts: quantitative continuous. Explanatory: (1) Side: categorical nominal with two levels: left and right. Left was set as reference. | Nleft = 50 Nright = 40 | Linear Mixed Model Regression Side Coef. = 0.071 z = 1.017 P-value = 0.31 TOST Bound = +- 0.28 p-value = 0.044 | Side IC95% = [-0.066, 0.207] |
| 2G right | Hedges’ g effect size for number of cycles. | Effect size difference right minus left. | Nleft = 50 Nright = 40 | Permutation t-test Difference = 0.233 P-value = 0.28 | IC95% = [-0.168, 0.627] |
| 3C | Phase Locking Value (R) for ripple-filtered phase difference between ipsi- and contra-lateral electrodes. | Response: Phase Locking Value (R): quantitative continuous. Explanatory: (1) Side: categorical nominal with two levels: ipsi- and contra-lateral. Ipsi was set as reference. | Nipsi = 220 Ncontra = 245 | Linear Mixed Model Regression Side Coef. = -0.445 z = -44.797 P-value ~= 0 Coef. in % of intercept: -84.6 % | Side IC95% = [-0.464, -0.425] |
| 3D | Phase Locking Value (R) for ripple-filtered phase difference between ipsi- and contra-lateral electrodes across shanks distances. | Response: Phase Locking Value (R): quantitative continuous. Explanatory: (1) Side: categorical nominal with two levels: ipsi- and contra-lateral. Ipsi was set as reference. (2) Distance: quantitative discrete for distances between electrodes, from 0 mm to 1200 mm. | Nipsi = 220 Ncontra = 245 | Linear Mixed Model Regression Side Coef. = -0.629 z = -44.497 P-value ~= 0 Distance Coef. = -0.076 z = -18.700 P-value = 4.94 x 10-78 Side x Distance Coeff = 0.077 z = 14.516 P-value = 9.64 x 10-48 | Side IC95% = [-0.656, -0.601] Distance IC95% = [-0.084, -0.068] Side x Distance IC95% = [0.067, 0.087] |
| 3D ipsi | Phase Locking Value (R) for ripple-filtered phase difference for ipsi-lateral electrodes across shanks distances. | Response: Phase Locking Value (R): quantitative continuous. Explanatory: (1) Distance: quantitative discrete for distances between ipsi-lateral electrodes, from 0 mm to 1200 mm. | Nipsi = 220 | Linear Mixed Model Regression Distance Coef. = -0.076 z = -13.578 P-value = 5.4 x 10-42 Coef. in % of intercept: -10.73 % | Distance IC95% = [-0.087, -0.065] |
| 3D contra | Phase Locking Value (R) for ripple-filtered phase difference for contra-lateral electrodes across shanks distances. | Response: Phase Locking Value (R): quantitative continuous. Explanatory: (1) Distance: quantitative discrete for distances between contra-lateral electrodes, from 0 mm to 1200 mm. | Ncontra = 245 | Linear Mixed Model Regression Distance Coef. = -0.001 z = -1.939 P-value = 0.053 Coef. in % of intercept: -1.56 % | Distance IC95% = [-0.003, 0.000] |
| 3F | Cross-correlation peak of ripple amplitude between ipsi- and contra-lateral electrodes. | Response: Amplitude peaks: quantitative continuous. Explanatory: (1) Side: categorical nominal with two levels: ipsi- and contra-lateral. Ipsi was set as reference. | Nipsi = 220 Ncontra = 245 | Linear Mixed Model Regression Side Coef. = -2.71 x 106 z = -49.477 P-value ~= 0 Coef. in % of intercept: -89.4 % | Side IC95% = [-2.8, -2.6] x 106 |
| 3G | Cross-correlation peak of ripple amplitude between ipsi- and contra-lateral electrodes. | Response: Amplitude peaks. quantitative continuous. Explanatory: (1) Side: categorical nominal with two levels: ipsi- and contra-lateral. Ipsi was set as reference. (2) Distance: quantitative discrete for distances between electrodes, from 0 mm to 1200 mm. | Nipsi = 220 Ncontra = 245 | Linear Mixed Model Regression Side Coef. = -3.72 x 106 z = -47.3 P-value ~= 0 Distance Coef. = -0.42 x 106 z = -18.302 P-value = 8 x 10-75 Side x Distance Coeff = 0.42 x 106 z = 14.339 P-value = 1.2 x 10-46 | Side IC95% = [-3.9, -3.6] x 106 Distance IC95% = [-0.46, -0.37] x 106 Side x Distance IC95% = [0.36, 0.48] x 106 |
| 3G ipsi | Cross-correlation peak of ripple-filtered signal between ipsi-lateral electrodes. | Response: Cross-correlation peak: quantitative continuous. Explanatory: (1) Distance: quantitative discrete for distances between electrodes, from 0 mm to 1200 mm. | Nipsi = 220 | Linear Mixed Model Regression Distance Coef. = -0.41 x 106 z = -14.257 P-value = 4.03 x 10-46 Coef. in % of intercept: -10.23 % | Distance IC95% = [-0.46, -0.35] x 106 |
| 3G contra | Cross-correlation peak of ripple-filtered signal between contra-lateral electrodes. | Response: Cross-correlation peak: quantitative continuous. Explanatory: (1) Distance: quantitative discrete for distances between electrodes, from 0 mm to 1200 mm. | Ncontra = 245 | Linear Mixed Model Regression Distance Coef. = -0.0094 x 106 z = -2.749 P-value = 0.006 Coef. in % of intercept: -2.44 % | Distance IC95% = [-0.016, -0.027] x 106 |
| Supp 3A | Phase Locking Value (R) for ripple-filtered phase difference during ripple events between ipsi- and contra-lateral electrodes. | Response: Phase Locking Value (R): quantitative continuous. Explanatory: (1) Side: categorical nominal with two levels: ipsi- and contra-lateral. Ipsi was set as reference. | Nipsi = 220 Ncontra = 245 | Linear Mixed Model Regression Side Coef. = -0.568 z = -45.370 P-value ~= 0 Coef. in % of intercept: -83.9 % | Side IC95% = [-0.592, -0.543] |
| Supp 3B | Phase Locking Value (R) for ripple-filtered phase difference during ripple events between ipsi- and contra-lateral electrodes across shanks distances. | Response: Phase Locking Value (R): quantitative continuous. Explanatory: (1) Side: categorical nominal with two levels: ipsi- and contra-lateral. Ipsi was set as reference. (2) Distance: quantitative discrete for distances between electrodes, from 0 mm to 1200 mm. | Nipsi = 220 Ncontra = 245 | Linear Mixed Model Regression Side Coef. = -0.787 z = -43.025 P-value ~= 0 Distance Coef. = -0.093 z = -17.649 P-value = 1.03 x 10-69 Side x Distance Coeff = 0.091 z = 13.278 P-value = 3.12 x 10-40 | Side IC95% = [-0.822, -0.751] Distance IC95% = [-0.104, -0.083] Side x Distance IC95% = [0.078, 0.105] |
| Supp 3B ipsi | Phase Locking Value (R) for ripple-filtered phase difference during ripple events for ipsi-lateral electrodes across shanks distances. | Response: Phase Locking Value (R): quantitative continuous. Explanatory: (1) Distance: quantitative discrete for distances between ipsi-lateral electrodes, from 0 mm to 1200 mm. | Nipsi = 220 | Linear Mixed Model Regression Distance Coef. = -0.091 z = -14.434 P-value = 3.17 x 10-47 Coef. in % of intercept: -10.25 % | Distance IC95% = [-0.104, -0.079] |
| Supp 3B contra | Phase Locking Value (R) for ripple-filtered phase difference during ripple events for contra-lateral electrodes across shanks distances. | Response: Phase Locking Value (R): quantitative continuous. Explanatory: (1) Distance: quantitative discrete for distances between contra-lateral electrodes, from 0 mm to 1200 mm. | Ncontra = 245 | Linear Mixed Model Regression Distance Coef. = -0.006 z = -5.346 P-value = 9 x 10-8 Coef. in % of intercept: -4.83 % | Distance IC95% = [-0.009, -0.004] |
| 4C | Correlation Coefficient (r) for instantaneous ripple amplitude between ipsi- and contra-lateral electrodes. | Response: Person’s Correlation Coefficient (r): quantitative continuous. Explanatory: (1) Side: categorical nominal with two levels: ipsi- and contra-lateral. Ipsi was set as reference. | Nipsi = 220 Ncontra = 245 | Linear Mixed Model Regression Side Coef. = -0.099 z = -13.871 P-value = 9.5 x 10-44 Coef. in % of intercept: -11.2% | Side IC95% = [-0.113, -0.085] |
| 4D | Correlation Coefficient (r) for instantaneous ripple amplitude between ipsi- and contra-lateral electrodes across shanks distances. | Response: Person’s Correlation Coefficient (r): quantitative continuous. Explanatory: (1) Side: categorical nominal with two levels: ipsi- and contra-lateral. Ipsi was set as reference. (2) Distance: quantitative discrete for distances between electrodes, from 0 mm to 1200 mm. | Nipsi = 220 Ncontra = 245 | Linear Mixed Model Regression Side Coef. = -0.144 z = -11.420 P-value = 3.3 x 10-30 Distance Coef. = -0.027 z = -7.486 P-value = 7.1 x 10-14 Side x Distance Coeff = 0.017 z = 3.684 P-value = 2.3 x 10-4 | Side IC95% = [-0.169, -0.119] Distance IC95% = [-0.034, -0.020] Side x Distance IC95% = [0.008, 0.027] |
| 4D ipsi | Correlation Coefficient (r) for instantaneous ripple amplitude between ipsi-lateral electrodes across shanks distances. | Response: Person’s Correlation Coefficient (r): quantitative continuous. Explanatory: (1) Distance: quantitative discrete for distances between ipsi-lateral electrodes, from 0 mm to 1200 mm. | Nipsi = 220 | Linear Mixed Model Regression Distance Coef. = -0.028 z = -7.671 P-value = 1.7 x 10-14 Coef. in % of intercept: -3 % | Distance IC95% = [-0.036, -0.021] |
| 4D contra | Correlation Coefficient (r) for instantaneous ripple amplitude between contra-lateral electrodes across shanks distances. | Response: Person’s Correlation Coefficient (r): quantitative continuous. Explanatory: (1) Distance: quantitative discrete for distances between contra-lateral electrodes, from 0 mm to 1200 mm. | Ncontra = 245 | Linear Mixed Model Regression Distance Coef. = -0.010 z = -3.927 P-value = 8.6 x 10-5 Coef. in % of intercept: -1.3 % | Distance IC95% = [-0.015, -0.005] |
| 4F | Cross-correlation peak of instantaneous ripple amplitude between ipsi- and contra-lateral electrodes across shanks distances. | Response: Cross-correlation peak: quantitative continuous. Explanatory: (1) Side: categorical nominal with two levels: ipsi- and contra-lateral. Ipsi was set as reference. | Nipsi = 220 Ncontra = 245 | Linear Mixed Model Regression Side Coef. = -0.95 x 106 z = -28.667 P-value = 9.9 x 10-181 Coef. in % of intercept: -11.9% | Side IC95% = [-1.01, -0.88] x 106 |
| 4G | Cross-correlation peak of instantaneous ripple amplitude between ipsi- and contra-lateral electrodes across shanks distances. | Response: Cross-correlation peak: quantitative continuous. Explanatory: (1) Side: categorical nominal with two levels: ipsi- and contra-lateral. Ipsi was set as reference. (2) Distance: quantitative discrete for distances between electrodes, from 0 mm to 1200 mm. | Nipsi = 220 Ncontra = 245 | Linear Mixed Model Regression Side Coef. = -1.4 x 106 z = -27.267 P-value = 1.04 x 10-163 Distance Coef. = -0.22 x 106 z = -14.495 P-value = 1.31 x 10-47 Side x Distance Coeff = 0.19 x 106 z = 9.672 P-value = 4 x 10-22 | Side IC95% = [-1.5, -1.3] x 106 Distance IC95% = [-0.24, -0.19] x 106 Side x Distance IC95% = [0.15, 0.22] x 106 |
| 4G ipsi | Cross-correlation peak of instantaneous ripple amplitude between ipsi-lateral electrodes across shanks distances. | Response: Cross-correlation peak: quantitative continuous. Explanatory: (1) Distance: quantitative discrete for distances between ipsi-lateral electrodes, from 0 mm to 1200 mm. | Nipsi = 220 | Linear Mixed Model Regression Distance Coef. = -0.22 x 106 z = -12.765 P-value = 2.6 x 10-37 Coef. in % of intercept: -2.58 % | Distance IC95% = [-0.25, -0.19] x 106 |
| 4G contra | Cross-correlation peak of instantaneous ripple amplitude between contra-lateral electrodes across shanks distances. | Response: Cross-correlation peak: quantitative continuous. Explanatory: (1) Distance: quantitative discrete for distances between contra-lateral electrodes, from 0 mm to 1200 mm. | Ncontra = 245 | Linear Mixed Model Regression Distance Coef. = -0.031 x 106 z = -3.575 P-value = 3.5 x 10-4 Coef. in % of intercept: -0.44 % | Distance IC95% = [-0.047, -0.0140] x 106 |
| 4 Supp 1 A | Phase Locking Value (R) for ripple-filtered phase difference between ipsi- and contra-lateral electrodes. Averaged across single sessions. | Response: Phase Locking Value (R): quantitative continuous. Explanatory: (1) Side: categorical nominal with two levels: ipsi- and contra-lateral. | Nipsi = 8 Ncontra = 8 | Paired T-Test R difference (ipsi - contra) = 0.44 t-value = 18.81 p-value = 2.98 x 10-7 dof = 7 | State IC95% = [0.38, 0.49] |
| 4 Supp 1 B | Correlation Coefficient (r) for instantaneous ripple amplitude between ipsi- and contra-lateral electrodes. Averaged across single sessions. | Response: Person’s Correlation Coefficient (r): quantitative continuous. Explanatory: (1) Side: categorical nominal with two levels: ipsi- and contra-lateral. | Nipsi = 8 Ncontra = 8 | Paired T-Test r difference (ipsi - contra) = 0.1 t-value = 4.3 p-value = 0.0035 dof = 7 | State IC95% = [0.048, 0.166] |
| 4 Supp 2 A left | Phase Locking Value (R) for ripple-filtered phase difference between ipsi- and contra-lateral electrodes. | Response: Phase Locking Value (R): quantitative continuous. Explanatory: (1) Side: categorical nominal with two levels: ipsi- and contra-lateral. Ipsi was set as reference. | Nipsi = 122 Ncontra = 121 | Linear Mixed Model Regression Side Coef. = -0.331 z = -22.291 P-value = 4.47 x 10-110 | Side IC95% = [-0.360, -0.302] |
| 4 Supp 2 A right | Correlation Coefficient (r) for instantaneous ripple amplitude between ipsi- and contra-lateral electrodes. | Response: Person’s Correlation Coefficient (r): quantitative continuous. Explanatory: (1) Side: categorical nominal with two levels: ipsi- and contra-lateral. Ipsi was set as reference. | Nipsi = 122 Ncontra = 121 | Linear Mixed Model Regression Side Coef. = -0.095 z = -10.05 P-value = 9.19 x 10-24 | Side IC95% = [-0.113, -0.076] |
| 4 Supp 2 B | Hedges’ g effect sizes for phase and amplitude coupling. | Left: Hedges’ g effect sizes (ipsi - contra) for Phase and Amplitude separately. Right: Hedges’ g effect size difference for Phase and Amplitude effect sizes (deltas’ g). Positive values indicate a higher effect size for Phase (i.e., a higher difference between ipsi- and contra-lateral phase coupling metric than amplitude coupling metric). | Nipsi = 122 Ncontra = 121 | Permutation t-test Phase Difference = 2.79 P-value = 0.0002 Amplitude Difference = 1.18 P-value = 0.0002 Deltas’ g (Phase - Amplitude) Difference = 1.61 P-value ~= 0 | Phase IC95% = [2.48, 3.16] Amplitude IC95% = [0.86, 1.57] Phase - Amplitude IC95% = [1.13, 2.12] |
| 5A | Hedges’ g effect sizes for Figure 3C, phase-phase coupling (Phase), measured as Phase Locking Value, and Figure 4C, amplitude – amplitude (Amplitude) coupling, measured as Pearson's Correlation Coefficient. | Left: Hedges’ g effect sizes (ipsi - contra) for Phase and Amplitude separately. Right: Hedges’ g effect size difference for Phase and Amplitude effect sizes (deltas’ g). Positive values indicate a higher effect size for Phase (i.e., a higher difference between ipsi- and contra-lateral phase coupling metric than amplitude coupling metric). | Nipsi = 220 Ncontra = 245 | Permutation t-test Phase Difference = 3.99 P-value ~= 0 Amplitude Difference = 0.859 P-value ~= 0 Deltas’ g (Phase - Amplitude) Difference = 3.2 P-value ~= 0 | Phase IC95% = [3.44, 4.48] Amplitude IC95% = [0.62, 1.05] Phase - Amplitude IC95% = [2.94, 3.48] |
| 5B | Paired Hedges’ g effect sizes differences (phase - amplitude) for each session separately from Figure 5A. Figure shows the paired difference between phase and amplitude. | Response: Hedges’ g effect sizes differences: quantitative continuous. Explanatory: (1) Analysis type: categorical nominal with two levels: phase and amplitude. | Nphase = 8 Namplitude = 8 | Paired T-test Analysis type Mean difference = 1.765 t – value = 4.58 P-value = 0.00254 | Analysis type IC95% = [1.11, 2.49] |
| 5C | Hedges’ g effect sizes for Figure 3F, phase-phase coupling (Phase), measured as cross-correlation peak value for ripple-filtered signal, and Figure 4F, amplitude – amplitude (Amplitude) coupling, measured as cross-correlation peak value for ripple instantaneous amplitude. | Left: Hedges’ g effect sizes (ipsi - contra) for Phase and Amplitude separately. Right: Hedges’ g effect size difference for Phase and Amplitude effect sizes (deltas’ g). Positive values indicate a higher effect size for Phase (i.e., a higher difference between ipsi- and contra-lateral phase coupling metric than amplitude coupling metric). | Nipsi = 220 Ncontra = 245 | Permutation t-test Phase Difference = 4.31 P-value ~= 0 Amplitude Difference = 2.07 P-value ~= 0 Deltas’ g (Phase - Amplitude) Difference = 3.28 P-value ~= 0 | Phase IC95% = [3.68, 4.91] Amplitude IC95% = [1.76, 2.32] Phase - Amplitude IC95% = [3.01, 3.55] |
| 5D | Paired Hedges’ g effect sizes differences (phase - amplitude) for each session separately from Figure 5C. Figure shows the paired difference between phase and amplitude. | Response: Hedges’ g effect sizes differences: quantitative continuous. Explanatory: (1) Analysis type: categorical nominal with two levels: phase and amplitude. | Nphase = 8 Namplitude = 8 | Paired T-test Analysis type Mean difference = 2.757 t-value = 3.94 P-value = 0.0056 | Analysis type IC95% = [1.419, 3.957] |
| 5 Supp 1 A left | Ripple abundance (event counts per second) across states. | Response: Counts per second: quantitative continuous. Explanatory: (1) State: categorical nominal with two levels: pre- and post-maze. Pre was set as reference. | Npre = 90 Npost = 90 | Linear Mixed Model Regression State Coef. = 0.039 z = 6.861 P-value = 6.85 x 10-12 | State IC95% = [0.028, 0.050] |
| 5 Supp 1 A right | Hedges’ g effect size for ripple counts. | Effect size difference post minus pre. | Npre = 90 Npost = 90 | Permutation t-test Difference = 1.14 P-value = 0.0002 | IC95% = [0.84, 1.53] |
| 5 Supp 1 B | Average ripple abundance (event counts per second) across states. | Response: Counts per second: quantitative continuous. Explanatory: (1) State: categorical nominal with two levels: pre- and post-maze. Pre was set as reference. | Npre = 8 Npost = 8 | Paired T-test counts difference (post – pre) = 0.038 t-value = 5.80 p-value = 0.00066 dof = 7 | IC95% = [0.022, 0.053] |
| 5 Supp 1 C | Phase Locking Value (R) for ripple-filtered phase difference between ipsi- and contra-lateral electrodes across states. | Response: Phase Locking Value (R): quantitative continuous. Explanatory: (1) Side: categorical nominal with two levels: ipsi- and contra-lateral. Ipsi was set as reference. (2) State: categorical nominal with two levels: pre- and post-maze. Pre was set as reference. | Nipsi = 220 Ncontra = 245 | Linear Mixed Model Regression Side Coef. = -0.444 z = -44.107 P-value ~= 0 State Coef. = -0.010 z = -0.938 P-value = 0.348 Side x State Coeff = 0.004 z = 0.257 P-value = 0.797 | Side IC95% = [-0.463, -0.424] State IC95% = [-0.030, 0.011] Side x State IC95% = [-0.024, 0.031] |
| 5 Supp 1 D | Correlation Coefficient (r) for instantaneous ripple amplitude between ipsi- and contra-lateral electrodes across states (pre- and post-maze exposition). | Response: Person’s Correlation Coefficient (r): quantitative continuous. Explanatory: (1) Side: categorical nominal with two levels: ipsi- and contra-lateral. Ipsi was set as reference. (2) State: categorical nominal with two levels: pre- and post-maze. Pre was set as reference. | Nipsi = 220 Ncontra = 245 | Linear Mixed Model Regression Side Coef. = -0.1 z = -13.247 P-value = 4.69 x 10-40 State Coef. = -0.001 z = -0.108 P-value = 0.914 Side x State Coeff = 0.011 z = 1.026 P-value = 0.305 | Side IC95% = [-0.116, -0.086] State IC95% = [-0.016, 0.014] Side x State IC95% = [-0.010, 0.032] |
| 5 Supp 1 E | Phase Locking Value (R) for ripple-filtered phase difference between ipsi- and contra-lateral electrodes. Averaged across single sessions. | Response: Phase Locking Value (R): quantitative continuous. Explanatory: (1) Side: categorical nominal with two levels: ipsi- and contra-lateral. Ipsi was set as reference. (2) State: categorical nominal with two levels: pre- and post-maze. Pre was set as reference. | Nipsi = 8 Ncontra = 8 | Linear Mixed Model Regression Side Coef. = -0.438 z = -23.335 P-value = 1.96 x 10-120 State Coef. = -0.010 z = -0.536 P-value = 0.592 Side x State Coeff = 0.004 z = 0.154 P-value = 0.878 Post-hoc pairwise t-tests (Benjamini–Hochberg FDR at 0.05) comparisons show that only the pre-post comparison within either ipsi (ipsi_post vs ipsi_pre) or contralateral (contra_post vs contra_pre) was non‑significant (p‑adj = 0.22 and 0.16, respectively), whereas all other pairwise comparisons were statistically significant (p‑adj < 0.05). | Side IC95% = [-0.475, -0.402] State IC95% = [-0.047, 0.027] Side x State IC95% = [-0.048, 0.056] |
| 5 Supp 1 F | Correlation Coefficient (r) for instantaneous ripple amplitude between ipsi- and contra-lateral electrodes. Averaged across single sessions. | Response: Person’s Correlation Coefficient (r): quantitative continuous. Explanatory: (1) Side: categorical nominal with two levels: ipsi- and contra-lateral. Ipsi was set as reference. (2) State: categorical nominal with two levels: pre- and post-maze. Pre was set as reference. | Nipsi = 8 Ncontra = 8 | Linear Mixed Model Regression Side Coef. = -0.108 z = -5 P-value = 5.73 x 10-7 State Coef. = -0.004 z = -0.187 P-value = 0.851 Side x State Coeff = 0.008 z = 0.278 P-value = 0.781 Post-hoc pairwise t-tests (Benjamini–Hochberg FDR at 0.05) comparisons show that only the pre-post comparison within either ipsi (ipsi_post vs ipsi_pre) or contralateral (contra_post vs contra_pre) was non‑significant (p‑adj = 0.78 and 0.73, respectively), whereas all other pairwise comparisons were statistically significant (p‑adj < 0.05). | Side IC95% = [-0.15, -0.066] State IC95% = [-0.046, 0.038] Side x State IC95% = [-0.051, 0.068] |
| 6A | Ripple coincidence between ipsi- and contra-lateral electrodes measured as the proportion of overlapping ripple events in 5, 50 of 100 ms bins. | Response: Proportion (%): quantitative continuous. Explanatory: (1) Side: categorical nominal with two levels: ipsi- and contra-lateral. Ipsi was set as reference. (2) Bin size (ms): quantitative discrete with values 5, 50 and 100 ms. | Nipsi = 220 Ncontra = 245 | Linear Mixed Model  Regression Side Coef. = -0.228 z = -21.495 P-value = 1.7 x 10-102 Bin size Coef. = 3.2 x 10-3 z = 26.674 P-value = 9.4 x 10-157 Side vs Bin size Coef. = 1.4 x 10-3 z = 8.815 P-value = 1.2 x 10-18 Tukey HSD (FWER = 0.05) post-hoc pairwise comparisons show significant differences between all conditions. | Side IC95% = [-0.248, -0.207] Bin size IC95% = [2.9, 3.4] x 10-3 Side x  Bin size IC95% = [1.1, 1.8] x 10-3 |
| 6B | Hedges’ g effect sizes for ripple coincidences. | Response: Hedges’ g effect sizes: quantitative continuous. Explanatory: (1) Side: categorical nominal with two levels: ipsi- and contra-lateral. Difference is shown as Ipsi minus Contra. (2) Bin size (ms): categorical ordinal with values 5, 50 and 100 ms bins. | Nipsi = 220 Ncontra = 245 | Permutation t-test 5 ms Difference = 1.7 P-value ~= 0 50 ms Difference = 0.769 P-value ~= 0 100 ms Difference = 0.706 P-value ~= 0 | 5 ms IC95% = [1.41, 1.98] 50 ms IC95% = [0.528, 0.984] 100 ms IC95% = [0.465, 0.919] |
| 6C | Cross-correlation of binarized ripple events  between ipsi- and contra-lateral electrodes. | Response: Counts: quantitative discrete. Explanatory: (1) Side: categorical nominal with two levels: ipsi- and contra-lateral. Ipsi was set as reference. (2) Bin size (ms): quantitative discrete with values 5, 50 and 100 ms. | Nipsi = 220 Ncontra = 245 | Linear Mixed Model  Regression Side Coef. = -517.531 z = -24.762 P-value = 2.3 x 10-135 Bin size Coef. = 5.196 z = 22.137 P-value = 1.4 x 10-108 Side vs Bin size Coef. = 2.94 z = 9.1 P-value = 9 x 10-20 Tukey HSD (FWER = 0.05) post-hoc pairwise comparisons show significant differences between all conditions except one: “contra – 50 ms bin” vs “ipsi – 5 ms bin”. | Side IC95% = [-558, -477] Bin size IC95% = [4.7, 5.7] Side x  Bin size IC95% = [2.3, 3.6] |
| 6D | Hedges’ g effect sizes for ripple cross-correlations. | Response: Hedges’ g effect sizes: quantitative continuous. Explanatory: (1) Side: categorical nominal with two levels: ipsi- and contra-lateral. Difference is shown as Ipsi minus Contra. (2) Bin size (ms): categorical ordinal with values 5, 50 and 100 ms bins. | Nipsi = 220 Ncontra = 245 | Permutation t-test 5 ms Difference = 1.92 P-value ~= 0 50 ms Difference = 1.07 P-value ~= 0 100 ms Difference = 0.957 P-value ~= 0 | 5 ms IC95% = [1.64, 2.18] 50 ms IC95% = [0.804, 1.3] 100 ms IC95% = [0.699, 1.18] |
| 7G left | Phase Locking Value (R) of pyramidal cell spike phases during ipsi- and contralateral ripples. | Response: Phase Locking Value (R): quantitative continuous. Explanatory: (1) Side: categorical nominal with two levels: ipsi- and contra-lateral. Ipsi was set as reference. | Npyr = 559 | Linear Mixed Model  Regression Side Coef. = -0.155 z = -56.052 P-value ~= 0 | Side IC95% = [-0.16, -0.15] |
| 7G right | Firing rate difference of pyramidal cells inside versus outside ripple events during ipsi- and contralateral ripples. | Response: Firing rate difference (Hz): quantitative continuous. Explanatory: (1) Side: categorical nominal with two levels: ipsi- and contra-lateral. Ipsi was set as reference. | Npyr = 559 | Linear Mixed Model  Regression Side Coef. = -0.166 z = -1.233 P-value = 0.218 | Side IC95% = [-0.431,  0.098] |
| 7H left | Phase Locking Value (R) of interneurons spike phases during ipsi- and contralateral ripples. | Response: Phase Locking Value (R): quantitative continuous. Explanatory: (1) Side: categorical nominal with two levels: ipsi- and contra-lateral. Ipsi was set as reference. | Nint = 128 | Linear Mixed Model  Regression Side Coef. = -0.106 z = -19.525 P-value = 6.77 x 10-85 | Side IC95% = [-0.117, -0.096] |
| 7H right | Firing rate difference of interneurons inside versus outside ripple events during ipsi- and contralateral ripples. | Response: Firing rate difference (Hz): quantitative continuous. Explanatory: (1) Side: categorical nominal with two levels: ipsi- and contra-lateral. Ipsi was set as reference. | Nint = 128 | Linear Mixed Model  Regression Side Coef. = -1.191 z = -0.511 P-value = 0.610 | Side IC95% = [-5.76,  3.38] |
| 7I | Proportion of significant pyramidal cells coupled to ripple phase. | Response: Proportion: quantitative continuous. Explanatory: (1) Side: categorical nominal with two levels: ipsi- and contra-lateral. Ipsi was set as reference. | Npyr = 559 | Proportion Side Ipsi: 0.85 Contra: 0.047 Proportion difference (contra minus ipsi) Difference = -0.805 P-value ~= 0 | Side IC95% = [-0.839, -0.773] |
| 7J | Proportion of significant interneurons coupled to ripple phase. | Response: Proportion: quantitative continuous. Explanatory: (1) Side: categorical nominal with two levels: ipsi- and contra-lateral. Ipsi was set as reference. | Nint = 128 | Proportion Side Ipsi: 0.95 Contra: 0.66 Proportion difference (contra minus ipsi) Difference = -0.281 P-value ~= 0 | Side IC95% = [-0.375, -0.211] |
| 7A Supp 2 | Phase Locking Value (R) of pyramidal spike phases during ipsi- and contralateral ripples across shanks distances. | Response: Phase Locking Value (R): quantitative continuous. Explanatory: (1) Side: categorical nominal with two levels: ipsi- and contra-lateral. Ipsi was set as reference. (2) Distance: quantitative discrete for distances between electrodes, from 0 mm to 1200 mm. | Npyr = 5953 | Linear Mixed Model  Regression Side Coef. = -0.284 z = -80.813 P-value ~= 0 Distance Coef. = -0.070 z = -68.456 P-value ~= 0 Side vs Distance Coef. = 0.070 z = 48.027 P-value ~= 0 | Side IC95% = [-0.291, -0.277] Distance IC95% = [-0.072, -0.068] Side vs Distance IC95% = [0.067, 0.072] |
| 7A Supp 2 ipsi | Phase Locking Value (R) of pyramidal spike phases during ipsilateral ripples across shanks distances. | Response: Phase Locking Value (R): quantitative continuous. Explanatory: (1) Distance: quantitative discrete for distances between ipsilateral electrodes, from 0 mm to 1200 mm. | Npyr = 3026 | Linear Mixed Model  Regression Distance Coef. = -0.070 z = -49.427 P-value ~= 0 Coef. in % of intercept: -22.6 % | Distance IC95% = [-0.073, -0.067] |
| 7A Supp 2 contra | Phase Locking Value (R) of pyramidal spike phases during contralateral ripples across shanks distances. | Response: Phase Locking Value (R): quantitative continuous. Explanatory: (1) Distance: quantitative discrete for distances between contralateral electrodes, from 0 mm to 1200 mm. | Npyr = 2927 | Linear Mixed Model  Regression Distance Coef. = -0.0002 z = -0.878 P-value = 0.38 Coef. in % of intercept: -0.8 % | Distance IC95% = [-0.001, 0] |
| 7B Supp 2 | Phase Locking Value (R) of interneurons spike phases during ipsi- and contralateral ripples across shanks distances. | Response: Phase Locking Value (R): quantitative continuous. Explanatory: (1) Side: categorical nominal with two levels: ipsi- and contra-lateral. Ipsi was set as reference. (2) Distance: quantitative discrete for distances between electrodes, from 0 mm to 1200 mm. | Nint = 1348 | Linear Mixed Model  Regression Side Coef. = -1.93 z = -33.385 P-value = 2.25 x 10-244 Distance Coef. = -0.044 z = -26.723 P-value = 2.54 x 10-157 Side vs Distance Coef. = 0.044 z = 19.127 P-value = 1.5 x 10-81 | Side IC95% = [-0.204, -0.181] Distance IC95% = [-0.047, -0.041] Side vs Distance IC95% = [0.039, 0.048] |
| 7B Supp 2 ipsi | Phase Locking Value (R) of interneurons spike phases during ipsilateral ripples across shanks distances. | Response: Phase Locking Value (R): quantitative continuous. Explanatory: (1) Distance: quantitative discrete for distances between ipsilateral electrodes, from 0 mm to 1200 mm. | Nint = 686 | Linear Mixed Model  Regression Distance Coef. = -0.044 z = -19.332 P-value = 2.9 x 10-83 Coef. in % of intercept: -20.5 % | Distance IC95% = [-0.048, -0.039] |
| 7B Supp 2 contra | Phase Locking Value (R) of interneurons spike phases during contralateral ripples across shanks distances. | Response: Phase Locking Value (R): quantitative continuous. Explanatory: (1) Distance: quantitative discrete for distances between contralateral electrodes, from 0 mm to 1200 mm. | Nint = 662 | Linear Mixed Model  Regression Distance Coef. = -0.0003 z = -0.978 P-value = 0.328 Coef. in % of intercept: -1.34 % | Distance IC95% = [-0.001, 0.000] |
| 7C Supp 2 | Firing rate difference (firing rate inside – outside ripple events) of pyramidal neurons during ipsi- and contralateral ripples across shanks distances. | Response: Firing rate difference (Hz): quantitative continuous. Explanatory: (1) Side: categorical nominal with two levels: ipsi- and contra-lateral. Ipsi was set as reference. (2) Distance: quantitative discrete for distances between electrodes, from 0 mm to 1200 mm. | Npyr = 5953 | Linear Mixed Model  Regression Side Coef. = -0.134 z = -1.387 P-value = 0.165 Distance Coef. = -0.036 z = -1.3 P-value =  0.194 Side vs Distance Coef. = -0.038 z = -0.946 P-value =   0.344 | Side IC95% = [-0.323, 0.055] Distance IC95% = [-0.091, 0.019] Side vs Distance IC95% = [-0.116, 0.04] |
| 7D Supp 2 | Firing rate difference (firing rate inside – outside ripple events) of interneurons during ipsi- and contralateral ripples across shanks distances. | Response: Firing rate difference (Hz): quantitative continuous. Explanatory: (1) Side: categorical nominal with two levels: ipsi- and contralateral. Ipsi was set as reference. (2) Distance: quantitative discrete for distances between electrodes, from 0 mm to 1200 mm. | Nint = 1348 | Linear Mixed Model  Regression Side Coef. = -1.29 z = -0.757 P-value = 0.449 Distance Coef. = -0.232 z = -0.48 P-value =  0.631 Side vs Distance Coef. = 0.068 z = 0.101 P-value = 0.92 | Side IC95% = [-4.628, 2.05] Distance IC95% = [-1.180, 0.715] Side vs Distance IC95% = [-1.25, 1.386] |
| 7A Supp 2 and 7B Supp 2 ipsi | Phase Locking Value (R) difference between 0 and 200 mm shanks distances  for pyramidal and interneurons’ spike phases during ipsilateral ripples. | Response: Phase Locking Value (R) difference: quantitative continuous. Explanatory: (1) Neuron type: categorical nominal with two levels: pyramidal and interneuron. | Npyr = 526 Nint = 115 | Independent T-Test Pyr mean diff = 0.27 Int mean diff = 0.12 Group diff = 0.16 t-value = 11.9 p-value = 1.2 x 10-29 dof = 639 |  |
